# Challenges and Opportunities in Breast Cancer Care in Low-Resourced Countries, Jordan as An Example

**DOI:** 10.3390/cancers16091751

**Published:** 2024-04-30

**Authors:** Hikmat Abdel-Razeq, Asem Mansour

**Affiliations:** 1 Department of Internal Medicine, King Hussein Cancer Center, Amman 11941, Jordan; 2 School of Medicine, The University of Jordan, Amman 11942, Jordan; 3 Department of Radiology, King Hussein Cancer Center, Amman 11941, Jordan; amansour@khcc.jo

**Keywords:** breast cancer, Jordan, low-income countries, LIC, early detection, screening

## Abstract

**Simple Summary:**

As the most common cancer diagnosed worldwide, breast cancer treatment represents a great opportunity to set up processes to improve the integration of all elements of cancer control programs. The journey starts with prevention, screening, and early detection, and includes survivorship programs that manage late after-treatment effects and, when needed, hospice and palliative care. Active treatment, using cost-effective up-to-date anti-cancer therapy delivered through a multidisciplinary approach, is the main pillar of this process. In this review, we address issues related to breast cancer in Jordan, some of which may also exist in neighboring and other low-resourced countries, such as presentation at a younger age and with advanced-stage disease. The rising number of newly diagnosed patients and the financial impact of the many recently introduced immunotherapies and targeted and endocrine therapies pose a great burden on most health care systems.

**Abstract:**

Jordan is a relatively small country with a rapidly growing population and a challenged economy. Breast cancer is the most diagnosed cancer among women worldwide and also in Jordan. Though the age-standardized rate (ASR) of breast cancer incidence is still lower than that in Western societies, the number of newly diagnosed cases continues to increase, involving younger women, and new cases are usually detected at more advanced stages. Improvements in breast cancer care across the health care continuum, including early detection, prevention, treatment, and survivorship and palliative care, have become very visible, but may not match the magnitude of the problem. More organized, goal-oriented work is urgently needed to downstage the disease and improve awareness of, access to, and participation in early detection programs. The cost of recently introduced anti-cancer therapies poses a great challenge, but the impact of these therapies on treatment outcomes, including overall survival, is becoming very noticeable. Though the concept of a multidisciplinary approach to breast cancer treatment is often used at most health care facilities, its implementation in real practice varies significantly. The availability of breast reconstruction procedures, survivorship programs, germline genetic testing, counselling, and palliative care is improving, but these are not widely practiced. In this manuscript, we review the status of breast cancer in Jordan and highlight some of the existing challenges and opportunities.

## 1. Introduction

Breast cancer continues to be the most commonly diagnosed cancer in Jordan and worldwide. In 2020, around 2.3 million new breast cancer cases and over 650,000 related deaths were reported worldwide [1,2,3]. In Jordan, cancer continues to be the second-leading cause of death after cardiovascular disease; since our last report 5 years ago [4], breast cancer has become the second most common cause of cancer-related death after lung cancer [5,6]. Data on the clinical presentation, pathology, and treatment outcomes for breast cancer in the region continue to be limited, and most of the data come from retrospective analyses, primarily reflecting researchers’ own experience at the institutional, not national, level. Recruitment for clinical trials is very limited, and participants from our region are notably under-represented.

Jordan is a relatively small country with a growing population of over 11.5 million people; population growth is mostly driven by the influx of refugees from neighboring countries, and most of the population reside in or around the capital city of Amman [7]. Currently, less than 5% of the population are 65 years or older [8]. Given improving life expectancy, it is expected that the population pyramid of the country will shift to host a greater proportion of older people, which may increase the number of women at risk for breast cancer. 

Jordan continues to suffer from a poor economy and a high unemployment rate. With a gross domestic product (GDP) of USD 4311.0 per capita, and poor growth in economic indicators, the country was recently reclassified by the World Bank as a “lower middle-income country” (LMIC) [9]. 

In Jordan, cancer care is provided free-of-charge by the government at Ministry of Health (MOH) hospitals, military hospitals (Royal Medical Services), university hospitals, and the King Hussein Cancer Center (KHCC). Very few patients are treated at their own cost at private hospitals and clinics, and those that are treated in this way are mostly those seeking drugs that are not available at public hospitals, like some of the newest targeted drugs and immunotherapies.

## 2. Materials and Methods

Published data on cancer care, including breast cancer, were searched for using PubMed and Google Scholar using the following keywords: cancer, breast cancer, Jordan, low-income countries, and low-resourced countries. Data on breast cancer incidence and the number of diagnosed and reported cases were obtained from the latest annual report published by the Jordan Cancer Registry (JCR), which is a population-based cancer registry that was established in 1996 by the Jordan MOH [6]. To ensure accuracy and comprehensiveness, reporting is compulsory, and cases with a tissue diagnosis are traced using a unique national identification number assigned to diagnosed patients. We believe that the system implemented is excellent at capturing all cancer patients, regardless of where they receive their diagnosis. However, the current national registry suffers from many problems and challenges that limit its expected benefits. For example, there is still a significant delay in the reporting of annual data, with a current lag period of 4–5 years; the latest reported data are from 2019. Additionally, the JCR data lack information on the staging of reported cancers and, more importantly, do not offer treatment outcomes or survival data. Given such limitations, we used data from the KHCC registry, which is a hospital-based registry established in 2006 that collects hundreds of data items on demographics, pathological characteristics, disease stages, treatment outcomes, and survival relating to all fully treated patients at the institution. Currently, the registry hosts data on over 65,000 patients. To assess treatment outcomes, including overall survival (OS), we collected data on all consecutive patients with pathologically confirmed diagnoses of breast cancer included in the KHCC cancer registry. The vital status at the time of data collection and analysis was obtained using records maintained and updated by the Department of Civil Status (DCS), a governmental agency that issues all death certificates. To ensure the accuracy of the outcome data, the KHCC cancer registry is directly connected to the DCS. Overall survival was estimated from the date of diagnosis to the date of death from any cause. Survival curves were presented using the Kaplan–Meier method, and the significance of differences in median survival duration between groups was assessed using the log rank test. To study the impact of recently introduced clinical interventions, mostly new drugs, we compared OS data of two groups of breast cancer patients with metastatic breast cancer treated before and after 2015.

## 3. Breast Cancer Burden

**Figure 1:**
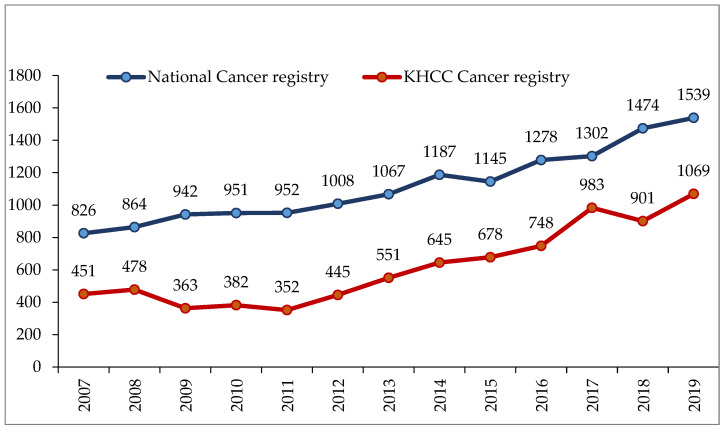
Number of new breast cancer cases diagnosed annually (KHCC: King Hussein Cancer Center).

Breast cancer is the leading cancer diagnosed in Jordan, representing over 18% of the total cancer burden in the country and close to 40% of all cancers diagnosed among women [10]. According to the latest report by the JCR, a total of 1539 breast cancer cases were diagnosed in the country in 2019, which represents an annual increase of almost 6% in the last decade; almost two-thirds of these patients were treated at KHCC (Figure 1). 

**Figure 2:**
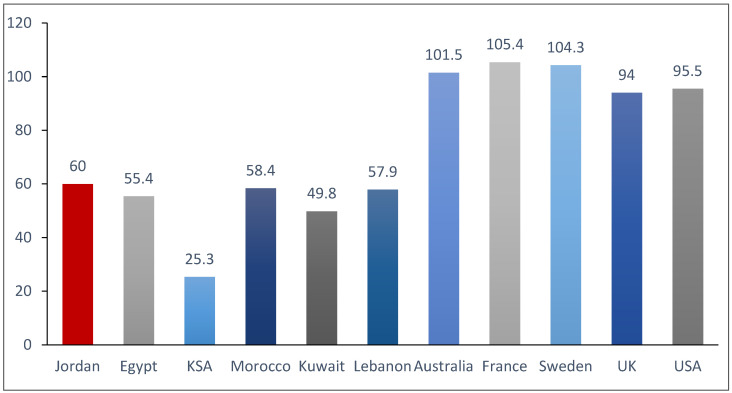
Estimated age-standardized incidence rate (ASR) of breast cancer in Jordan and some neighboring countries, compared to Western countries (per 100,000).

According to the latest Global Cancer Burden report issued by the International Agency for Research on Cancer (IARC) in 2022, the estimated number of breast cancer cases diagnosed in 2022 was 2598 cases, and the age-standardized incidence rate (ASR) was 60.0 per 100,000, which is similar to the estimated rates in the region, but significantly lower than estimates in Western societies in North America, Australia, and European countries, as illustrated in Figure 2 [11,12]. 

## 4. Screening and Early Detection

The majority of breast cancer cases in non-Western countries, including ours, are not screen-detected; most cases are instead diagnosed clinically with large tumors, and often with nodal involvement [13]. Fewer than one-third of women in Jordan present with localized disease at the time of diagnosis. Compared to rates lower than 6% in the Unites States and the United Kingdom, our data indicate that over 13% of our patients are diagnosed with metastatic disease at their first presentation [4].

Huge efforts have been made to enhance women’s awareness about the importance of mammography screenings to detect breast cancer at earlier stages, when chances of recovery are high, and the extent of surgical intervention is less intense. The Jordan Breast Cancer Program (JBCP) was established in 2007 as a national program led and supported by the King Hussein Cancer Foundation and Center, the MOH, and all health care sectors in the country [14]. The program coordinates breast cancer early detection efforts across the country, raises public awareness about breast cancer, and provides screening services. Due to these efforts, the proportion of breast cancer patients diagnosed with locally advanced or metastatic stages of the disease has significantly decreased. The JBCP is also heavily involved in capacity building by training radiology technicians and radiographers, installing mammography units across the country, and providing opportunities for central reading. Since its establishment, the JBCP has managed to install over thirty certified mammography units; twenty (two-thirds) of them are located outside the capital city of Amman. The program also provides mobile mammography units that can reach remote areas in the country. The units undergo regular certification by the Jordanian Health Care Accreditation Council (HCAC). More recently, the JBCP has just published its updated national guidelines on breast cancer early detection and screening, and the approach to the diagnostic work-up for women with positive findings [15]. The JBCP reiterated its previous stance by recommending starting mammography screenings at an earlier age (40 years old). In its suggested “in-progress” guidelines, the U.S. Preventive Services Task Force (USPSTF) is also suggesting an earlier start at age 40, instead of 50, as previously recommended [16]. However, two differences between these two guidelines exist: the USPSTF’s recommendations are for biennial, not annual, screening, and the USPSTF also recommends against mammography screening in women aged 75 years or older, as the current evidence is insufficient to assess the balance of benefits and harms of screening in this age group [17]. Table 1 summarizes the JBCP’s (national) screening recommendations and many international recommendations, including those of the USPSTF, the National Comprehensive Cancer Network (NCCN) [18], and the American Cancer Society (ACS) [19]. Despite all of these efforts and investments, only a small proportion of eligible women undergo mammography screening as recommended by the national guidelines.

## 5. Breast Cancer Prevention 

Systemic programs designed to help prevent breast cancer are lacking in low-resource countries, and Jordan is not an exception [20,21,22]. The introduction of a clinical genetic cancer program at KHCC represents the first effort in the country to explore the issue of hereditary breast cancer among women at risk. 

The use of chemopreventive medications, including selective estrogen receptor modulators (SERMs) like tamoxifen and raloxifene, and aromatase inhibitors (AI) like anastrozole and exemestane, is not practiced, though efforts to start a chemoprevention program are underway at KHCC [23,24,25].

The lack of a primary care practice in the country limits the efficiency of promoting lifestyle modification as a risk-reducing strategy. Smoking, high fat intake, obesity and lack of adequate exercise remain health care problems in the country that need to be addressed through a comprehensive program [26].

## 6. Germline Genetic Testing

Hereditary breast cancer accounts for 10% or more of all breast cancer burden worldwide [27]. With expanded indications and wider access to molecular testing, this proportion is expected to rise. Individuals with pathogenic, or likely pathogenic, variants of *BRCA1* and *BRCA2* are at higher risk of breast, ovarian, and pancreatic cancers, and possibly other forms of cancer, too [28,29]. 

Over the past two decades, several other moderately and highly penetrant cancer-susceptibility genes, like *PALB2*, *CHEK2*, and *ATM*, have been identified, highlighting the importance of integrating genetic counselling programs with routine clinical breast cancer care. In addition to its important role in preventing cancer in patients and their close family members, identification of pathogenic or likely pathogenic variants, at least in *BRAC1* and *BRCA2*, may inform therapeutic decisions in common cancers including breast, ovarian, prostate, and pancreatic cancers. Several randomized clinical trials have shown that the use of poly ADP ribose polymerase (PARP) inhibitors, such as olaparib (the OlympiAD trial) [30] or talazoparib (the EMBRACA trial) [31], has been associated with better progression-free survival (PFS) compared to single-agent palliative chemotherapy in patients with locally advanced or metastatic HER2-negative breast cancer with a pathogenic/likely pathogenic germline *BRCA1/2* mutation. Likewise, PARP inhibitors were also trialed in the treatment of patients with early-stage breast cancer of *BRCA1/2* pathogenic or likely pathogenic variants. In one study (the OlympiAD trial), olaparib was associated with significant improvement in both disease-free survival (DFS) and OS compared to the placebo [32].

We follow the NCCN [33] and other international guidelines [34], including the American Society of Clinical Oncology (ASCO) [35], for germline genetic testing. These guidelines are frequently updated, and not all physicians, including medical and surgical oncologists, remain familiar with such updates, a factor that may contribute to the lower referral rate of eligible patients for testing and counselling. Recently, the eligible age at breast cancer diagnosis to commence germline genetic testing was raised by the ASCO and the American Society of Surgical Oncology to 65 years [36,37], which will increase the proportion of eligible patients in societies like ours to over 90% of all breast cancer patients. We have recently completely one of the biggest germline genetic testing studies in the region, in which 3319 unselected Jordanian patients with any cancer (>50% were breast cancer) received germline genetic testing (GGT). In this study, and in many previous studies from our group [38], over 13% of Jordanian breast cancer patients tested as per the international guidelines had pathogenic or likely pathogenic variants [39].

However, genetic testing faces several challenges. First, compliance with testing guidelines is relatively low and varies across institutions, and when offered, patients may have to pay for the test, which can be a financial burden. Cost can also be a major issue for family members when cascade testing is requested. Second, trained and experienced genetic counselors do not exist in the country. To manage this problem, a genetic counselling training program was established at KHCC, allowing health care workers with different medical backgrounds, including molecular biology, nursing, and pharmacology, to be enrolled. In the meantime, much of the pre- and post-testing counselling is performed by primary oncologists themselves. Third, communication between family members about genetic test results is unexpectedly low; very few patients share the results of their genetic testing with their at-risk relatives, and that is reflected in the relatively low rate of cascade testing among family members [40]. Fourth, and most importantly, the cost of risk-reducing surgery like prophylactic mastectomy and oophorectomy might not be covered by public or private health insurance systems [41]. Social stigma, as a limiting factor against germline genetic testing, was not an issue that we frequently encountered [42].

## 7. Treatment of Breast Cancer across the Continuum

### 7.1. Medical Therapy

**Figure 3:**
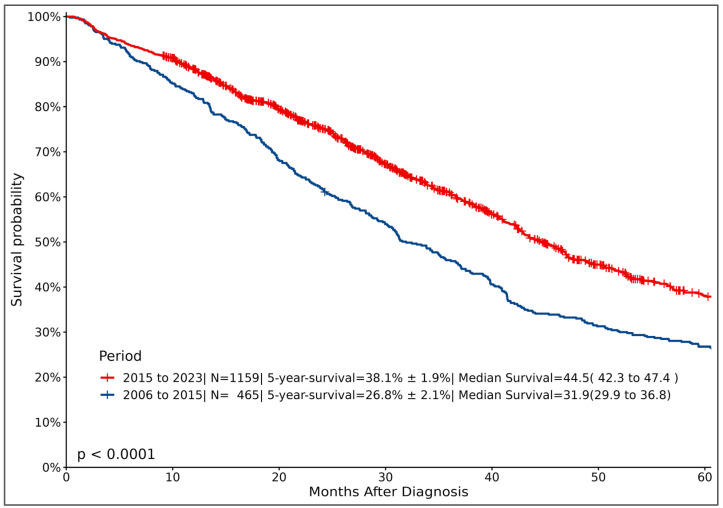
Overall survival (OS) of patients with metastatic breast cancer (MBC) by year of treatment.

The last decade has witnessed a revolution in the treatment of breast cancer across all stages, but especially for those with advanced-stage disease. Almost all chemotherapy drugs are available at major public hospitals, as well as in the private sector. Similarly, anti-HER2 drugs, including trastuzumab, pertuzumab [43], lapatinib [44], and ado-trastuzumab emtansine (T-DM1) [45], are widely available. However, almost all antibody drug conjugates (ADCs)—a class of targeted medications that combine monoclonal antibodies with cytotoxic payloads via a linker, including sacituzumab govitecan [46], trastuzumab duocarmazine [47], and trastuzumab deruxtecan (T-DXd) [48,49]—are not available at public hospitals or at KHCC. Many endocrine treatments, including drugs that modulate endocrine resistance, like CDK4/6 inhibitors (ribociclib [50], palbociclib [51], and abemaciclib [52]), were introduced relatively early, soon after their approval, for the treatment of HR-positive and HER2-negative metastatic breast cancer, but not in adjuvant settings for patients with early-stage disease. Phosphoinositide 3-kinase (PI3K) inhibitors (alpelisib [53], inavolisib [54]) are available as part of clinical trials at KHCC and local private hospitals, while immunotherapy drugs, like pembrolizumab [55]—which targets and blocks a protein called PD-1 on the surface of certain immune cells—are widely available and approved for both early-stage disease as part of neoadjuvant/adjuvant therapy and those with advanced-stage, triple-negative breast cancer. Capivasertib [56], an AKT inhibitor, was recently approved but is not available, while olaparib, a PARP inhibitor, was recently approved for breast cancer patients with pathogenic or likely pathogenic *BRCA1* or *BRCA2* variants. The effect of these drugs on treatment outcomes is very evident and can be better illustrated by reviewing the survival outcomes of patients with advanced-stage disease treated prior to the year 2015 and those after. Figure 3 clearly demonstrates the better OS of patients treated in recent years (median OS: 44.5 months) utilizing many of these recently introduced drugs, compared to 31.9 months (*p* < 0.0001) for those treated prior to 2015.

Cost, however, continues to be the major barrier that limits and delays the introduction of many new innovative anti-cancer therapies. At KHCC, the pharmacy and therapeutic (P&T) committee extensively and scientifically evaluates the cost-effectiveness of these drugs before their introduction to the hospital formulary. Such a process is not strictly adopted elsewhere in the country; however, similar and less extensive services were recently started at university hospitals, but not at MOH-run hospitals or those in the private sector.

### 7.2. Breast Surgical Oncology

Surgical treatment of breast cancer is widely available at most hospitals; however, very few hospitals have the capacity to practice and perform “surgical oncology” with proper techniques that ensure tumor resection with adequate margins, sentinel axillary lymph nodes, and oncoplastic procedures. One of the major problems encountered is the timing of surgery in relation to chemotherapy, when needed. The concept of neoadjuvant chemotherapy is not always followed. Unfortunately, it is not unusual for high-risk patients, including those with triple-negative or HER2-postive locally advanced disease, to undergo upfront surgery without the option of neoadjuvant therapy being discussed. Likewise, coordinating germline genetic testing with the type, extent, and timing of surgical intervention is not strictly followed outside of major institutions. Breast reconstruction surgery is unfortunately not covered by most private and public insurance systems. Most of the time, patients have to pay for the procedure, or at least contribute significantly to its cost. 

### 7.3. Radiation Therapy

Radiation therapy is widely available at almost all public-sector hospitals, and also at one private center. However, except for one location in the northern part of the country, all other centers are located within the capital city of Amman. Patients living in the southern region have to travel 3–4 h, sometimes daily, to receive radiation therapy. Psychosocial services do provide residential support for such patients and relatives while undergoing the planned treatment. It is the intention of KHCC to open a satellite facility in the southern region for chemotherapy infusion initially, then radiation therapy, to support patients living there. 

We believe the number of radiation therapy machines and associated logistics are just adequate for the country’s current needs; however, service expansion is underway at many facilities, including the KHCC, where two new accelerators, along with MRI-based simulation, will be added in the next few months. Advanced radiation therapy techniques and schedules are being offered to breast cancer patients, including the management of solitary metastasis. 

### 7.4. Palliative Care and Hospice

Many patients with breast cancer in our country present with advanced-stage disease, and a significant percentage of those who present with early-stage disease will progress despite the introduction of many new anti-cancer agents. Dealing with breast cancer patients when they reach a stage where the goal of treatment is only palliation requires extensive experience with supportive care, symptom control, pain management, and psychosocial issues. Palliative care, as an independent service, is limited to large centers. To advance this service further, hospice and home care services are urgently needed to complement inpatient medical care. In addition to enhancing patients’ quality of life and satisfaction, such services can minimize the cost of therapy by avoiding unnecessary emergency room visits, hospital admissions and unnecessary medical intervention. 

The King Hussein Cancer Center took the lead to structure palliative care and hospice as a distinct specialty, run by a specialized team of well-trained physicians, nurses, and pharmacists. Additionally, this service is fully integrated and linked to psychosocial programs with psychiatrists, psychologists, and social workers who attend to all of the needs of patients and their care givers. The palliative care program is fully accredited by the Jordanian Medical Council, the official accrediting body in the country, and graduates are granted a specialty board certification in palliative care. 

### 7.5. Breast Cancer Survivorship Program

Recent data have clearly shown that breast cancer mortality is declining. Since 1989, breast cancer mortality in the United States has declined by 43% [57]. However, racial disparity is still an issue, with many of the deaths being recorded among marginalized patients [58]. The recent introduction of tens of new endocrine therapies, mostly tackling resistance pathways, along with immunotherapy and antibody drug conjugates (ADCs), has resulted in declines in breast cancer-related mortality and will hopefully result in further declines at a much higher pace in the future. As such, more women will survive their disease, highlighting the importance of expanding and establishing new breast cancer survivorship programs. The American College of Surgeons (ACS) has released standards that include requirements that a comprehensive care summary and follow-up plan be provided to all individuals receiving curative intent therapy [59,60]. Issues related to bone health [61,62], fertility [63,64], sexual dysfunction [65,66], and surveillance for cancer recurrence or secondary malignancies are among the important issues addressed by breast cancer survivorship programs [67,68,69]. 

A national cancer survivorship program in Jordan does not currently exist. The only program in the country was established at KHCC in 2015 and started as a breast cancer survivorship program. The program focuses heavily on clinical care, along with all survivorship dimensions similar to those recommended by the ACS. More recently, the program has started to expand its the dimensions of its service to include research and education, too. Since its establishment, the program has seen and followed-up with over 3500 breast cancer survivors. The program is run by a trained family physician specialist and a nurse coordinator. 

Primary care medicine continues to be a problem in Jordan. Very few patients are followed-up with by their primary care physicians. Such a shortage is putting extraneous pressure on oncologists and cancer services/centers to continue following patients for an extended time, sometimes indefinitely. Additionally, oncologists are expected to take care of all patients’ medical problems and manage their comorbidities. 

## 8. Conclusions and Future Directions

Breast cancer continues to be the most commonly diagnosed cancer in Jordan. Giving its increasing incidence and the anticipated changes in the nation’s population structure, it is anticipated that breast cancer will continue to pose a national health care problem. Investment in breast cancer prevention programs and early detection to downstage the disease, improve access to comprehensive cancer care, and establish survivorship programs that minimize subsequent cancers and after-treatment consequences. Should help improve outcomes. 

Our insights presented in this review are not without limitations. Though we tried to look into breast cancer issues at a national level, the data, thoughts, and ideas presented represent mostly our own experience at a single institution, and might not precisely reflect the practice in the country at large. National data that addresses breast cancer care at all levels, from screening uptake to disease staging, pathology subtypes, treatment offered, and survival data, need to be organized and made available to researchers and decision-makers to help build a national strategy to tackle a potentially curable cancer that represents over 20% of the national cancer burden. 

Moreover, variations in local demographics and economics might affect the status of cancer care, for breast cancer in particular, in other resource-restricted countries. However, the thoughts, suggestions, and recommendations from practicing physicians and administrators that we presented in this work will hopefully help physicians and policymakers to improve breast cancer care. 

## Figures and Tables

**Table 1:** Summary of screening recommendations for breast cancer.

Age Group (Years)	Mammogram				Clinical Breast Exam			
	JBCP	USPSTF	NCCN	ACS	JBCP	USPSTF	NCCN	ACS
25–39	Not Rec.	Not Rec.	Not Rec.	Not Rec.	Annual	Additional potential benefit exists	Every 1–3 years	Not Rec.
40–74	Annual	Biennial	Annual with tomosynthesis	-Age 40–44: Women should have the opportunity to begin annual screening -Age 45–54: Annual -Age ≥55: Biennial	Annual	Additional potential benefit exists	Annual	Not Rec.
≥75	Annual	Not Rec. *	Annual ^#^	Biennial ^$^	Annual	Additional potential benefit exists	Annual	Not Rec.

JBCP: Jordan Breast Cancer Program; USPSTF: United States Preventive Services Task Force; NCCN: National Comprehensive Cancer Network; ACS: American Cancer Society; Not Rec.: Not recommended. * The USPSTF concludes that the current evidence is insufficient to assess the balance of benefits and harms of mammography screening in women aged 75 years or older. ^#^ The NCCN Panel has not established an upper age limit for screening. If a patient has severe comorbid conditions limiting life expectancy and no further intervention would occur based on the screening findings, then the patient should not undergo screening, regardless of age. ^$^ Women should continue mammography screening as long as their overall health is good, and they have a life expectancy of 10 years or longer.

## Data Availability

Data are contained within the article.
